# Morphological segmentation with tiling light sheet microscopy to quantitatively analyze the three-dimensional structures of spinal motoneurons

**DOI:** 10.1186/s13619-025-00231-3

**Published:** 2025-05-14

**Authors:** Huijie Hu, Dongyue Wang, Yanlu Chen, Liang Gao

**Affiliations:** 1 College of Life Sciences, Zhejiang University, Hangzhou, 310058 Zhejiang China; 2 Key Laboratory of Structural Biology of Zhejiang Province, School of Life Sciences, Westlake University, Hangzhou, 310024 Zhejiang China; 3 Westlake Laboratory of Life Sciences and Biomedicine, Hangzhou, 310024 Zhejiang China

**Keywords:** Tiling light sheet microscopy, Tissue clearing methods, Spinal motoneurons, 3D volumetric reconstruction, Soma size, Dendritic arborization

## Abstract

Spinal motoneurons control muscle fibers contraction and drive all motor behaviors in vertebrates. Although spinal motoneurons share the fundamental role of innervating muscle fibers, they exhibit remarkable diversity that reflects their specific identities. Defining the morphological changes during postnatal development is critical for elucidating this diversity. However, our understanding of the three-dimensional (3D) morphology of spinal motoneurons at these stages remains limited, largely due to the lack of high-throughput imaging tools. Using tiling light sheet microscopy combined with tissue clearing methods, we imaged motoneurons of the lateral and median motor column in the cervical and lumbar cord during postnatal development. By analyzing their soma size, we found that motoneurons innervating the upper limbs differentiate into two subpopulations with distinct soma size by postnatal day 14 (P14), while differentiation of motoneurons innervating the lower limbs is delayed. Furthermore, coupling adenovirus labeling with 3D volumetric reconstruction, we traced and measured the number and lengths of dendrites of flexor and extensor motoneurons in the lumbar cord, finding that the number of dendrites initially increases and subsequently declines as dendritic order rises. Together, these findings provide a quantitative analysis of the 3D morphological changes underlying spinal motoneuron diversity.

**Supplementary Information:**

The online version contains supplementary material available at 10.1186/s13619-025-00231-3.

## Background

All motor commands, whether voluntary or involuntary, are ultimately integrated by spinal motoneurons (SpMNs) (Arber and Costa 2018; Arber 2012). SpMNs receive neural projections from descending pathways, spinal interneurons, and peripheral sensory inputs (Côté et al. 2018; de Carvalho and Swash 2023), transmitting all signals to muscle fibers via neuromuscular junctions (Stifani 2014). Motor behaviors depend on the coordinate recruitment of different muscle groups, each activated by a specialized set of SpMNs (Agalliu et al. 2009). A distinct anatomical correlation exists between SpMNs and muscle fibers, enabling the temporal and spatial synchronization of muscle groups under the control of spMNs (Kernell 2006).

To facilitate the production of varying levels of force output in response to diverse movement scenarios, motor pools typically comprise three distinct types of SpMNs (Bączyk et al. 2022; Dasen 2022; Simon et al. 1996; Kernell 2006; Stifani 2014). Establishment of cellular diversity during development is essential for SpMNs to execute precise movements. In mature motor neuron pools, αMNs form exclusive connections with extrafusal muscle fibers to elicit muscle contractions, whereas γMNs—lacking 1a sensory presynaptic boutons—innervate only the intrafusal fibers within muscle spindles (Khan et al. 2022). In addition to their connectivity differences, these two populations also exhibit distinct electrophysiological characteristics (Manuel and Zytnicki 2019). Notably, αMNs are characterized by larger soma sizes, while γMNs tend to have smaller soma (Simon et al. 1996). This morphological and functional divergence underscores the importance of developing methods to quantitatively analyze changes in soma size during postnatal development, as such measurements are critical for understanding the functional differentiation of MNs. Over the past decades, transcription factor and epigenetic-mediated MN development has been extensively studied in vivo (Agalliu et al. 2009; Ashrafi et al. 2012; Liau et al. 2023; Mendelsohn et al. 2017; Dasen et al. 2003; Bulajić et al. 2020). However, anatomical studies of MNs during spinal cord development were limited to 2D histological assessment (Kanjhan et al. 2016a, b; Fukuda et al. 2020). It remains unclear how different types of MNs are organized within the spinal cord and how the spatial organization changes during development.

As regards imaging, several fluorescence microscopy techniques are available for obtaining volumetric data of whole tissues (Zhong et al. 2021; Pesce et al. 2022). However, while the acquisition speed is an inherent limitation for imaging large-volume tissues, tiling light sheet microscope (TLSM) overcomes this by enabling rapid acquisition of cleared samples. TLSM enables high throughput 3D imaging of centimeter-scale cleared and expanded biospecimens with spatial resolutions ranging from microns to sub-hundred nanometers (Chen et al. 2020). The combination of tissue clearing and TLSM techniques provided a powerful tool for investigating 3D morphological diversity and spatial organization of MNs during postnatal development.

MNs are organized hierarchically, with distinct columns aligned along the rostrocaudal axis of the spinal cord, each targeting specific peripheral structures (Tosney et al. 1995). The median motor column (MMC) and lateral motor column (LMC) neurons are essential for mice, as they coordinate axial muscle activity to maintain posture and balance while enabling precise limb movements critical for locomotion and other motor behaviors (Patani 2016; Nicolopoulos-Stournaras and Iles 1983; Gutman et al. 1993; Dasen 2022). Here, we conducted quantitative analysis of the changes in 3D soma size of MMC and LMC neurons in the cervical and lumbar cord at five ages (P1, P7, P14, P28 and P56).

In addition, dendrites integrate synaptic inputs while minimizing metabolic costs by extending to existing or potential synaptic targets, making the understanding of the dendritic arborization of spinal motoneurons essential (Stuart and Spruston 2015; Magee 2000). Sparse labeling is an important aspect of tissue preparation for dendrite tracing and analysis (Bloss et al. 2018; Lin et al. 2018). Over the last two decades, adenovirus (AdV) vectors have become powerful tools for labeling single neuron and treating diseases of central nervous system (CNS) (Salinas et al. 2009; Andrew Paul Tosolini and Morris 2016). In this study, we found that adenoviral injections produced robust fluorescent expression in MNs with high spatiotemporal resolution, effectively labeling retrogradely. We used AdV via intramuscular injection to label MNs innervating paired flexor and extensor muscles at P4, P14, and P56.

Accurate 3D quantification of soma and dendrites is essential for understanding the normal and pathological neuronal function of MNs. However, traditional quantification has relied on slow, labor-intensive methods like manual counting, which are low-throughput and unsuitable for large samples (Herculano-Houzel and Lent 2005; Fukuda et al. 2020). Specifically, manual editing will vary depending on the complexity of the image data. This article presented semi-automatic segmentation methods for objective morphological analysis by providing image acquisition parameters with Amira. These protocols will serve as a valuable reference for scientists aiming to quantify and characterize neural structures in the CNS.

Collectively, we imaged the MMC and LMC neurons in the cervical and lumbar cord during postnatal development as well as MNs inverting paired flexor and extensor muscles. We further presented protocols for 3D volumetric analysis of soma size and dendritic arborization. These morphological analyses offer valuable insights into the diversity of MNs during postnatal development.

## Results

### Soma segmentation with Amira

**Fig. 1:**
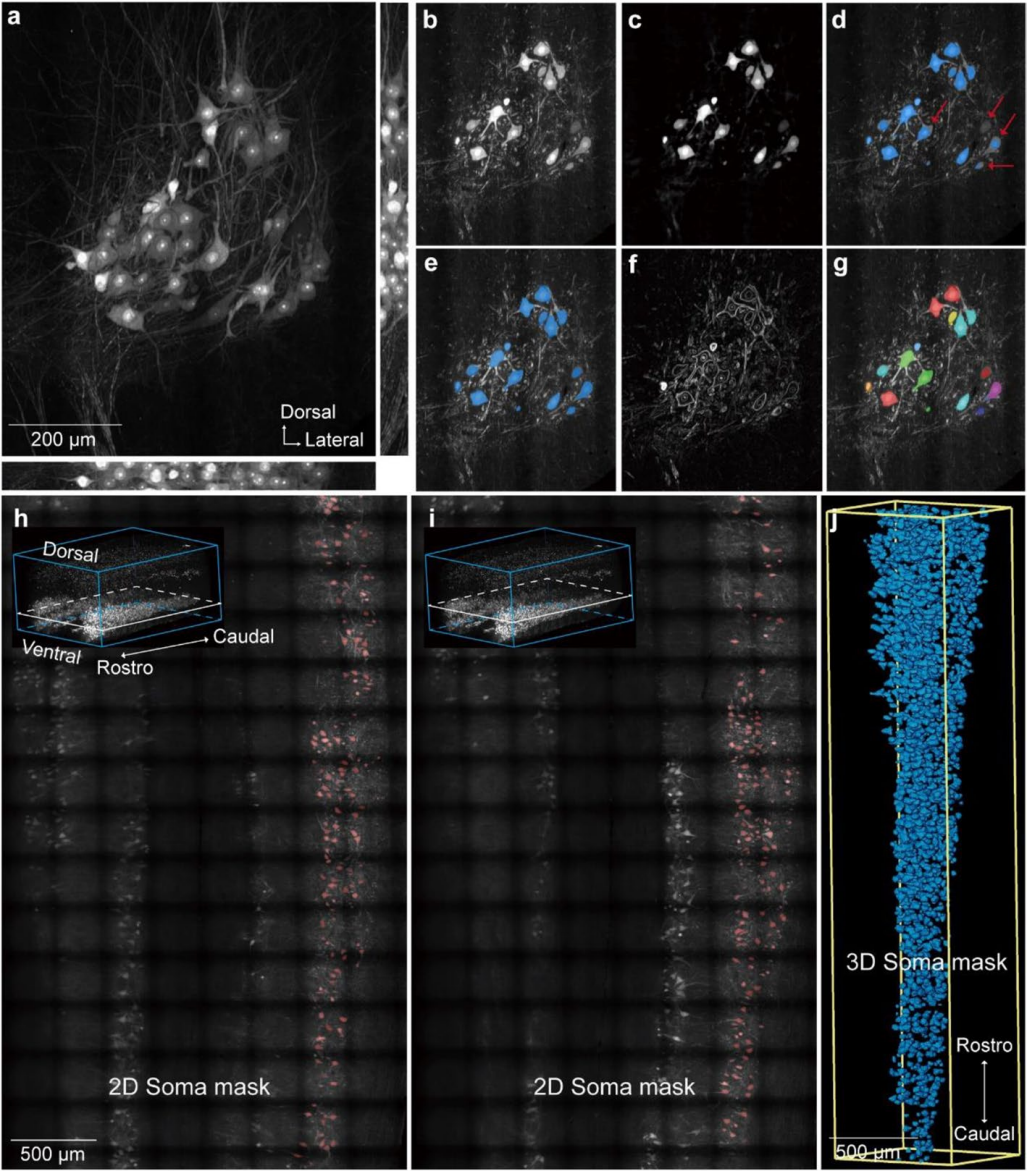
The semi-automatic cell segmentation workflow utilizing the deep learning algorithm. **a** Grayscale image of a small 3D block with a data size of approximately 30 MB, with a scale bar of 200 μm. **b** A single slice extracted from (**a**). **c** Grayscale image after processing with the *Median Filter* command. **d** Automatically extracted soma boundaries obtained using the *Hysteresis Thresholding* command. **e** Soma boundaries predicted by the deep learning-trained model, with a scale bar of 500 μm. **f** Grayscale image after the *Image Gradient* command processing. **g** Soma segmentation achieved through the *Marker-based Watershed inside Mask* command. **h**, **i** Soma boundaries in the 3D image predicted by the deep learning-trained model, with a scale bar of 500 μm. **j** 3D rendering of the soma boundaries, with a scale bar of 500 μm

The TLSM was employed in conjunction with the CUBIC-L tissue clearing method (Matsumoto et al. 2019) to image the spinal cord of ChAT-eGFP mice at five stages (P1, P7, P14, P28, and P56). A more detailed description of the alignment and operation of TLSM can be found in the previous publication (Feng et al. 2021). Register and merge the images from adjacent sample volumes with Amira (Feng et al. 2021). The high density of MNs in the spinal cord of young mice (e.g., at P1 and P7) presents a challenge for automated single-cell segmentation due to the minimal spacing between MNs. To address this issue, we developed a semi-automated soma segmentation workflow based on deep learning algorithms using the commercial software, Amira. All operations were performed using Amira commands. The *Extract Subvolume* command was used to extract a small image block with a data size of approximately 30 megabytes (Fig. 1a, b). Then, we used the *Median Filter* command to erode the signal in the dendrites and axons of MNs. The filter can remove tiny structures, like dendrites and axons, without affecting the boundaries of large components, such as somas (Fig. 1c). Next, the *Hysteresis Thresholding* command was used to convert the grayscale image into a binary image, represented by a blue mask (Fig. 1d). However, as shown by the red arrows in Fig. 1d, this process failed to accurately extract every soma. To enhance the accuracy of soma region extraction, a deep learning command (*DL Training-Segmentation 3D*) was employed. The parameter settings of deep learning command were presented in Table S1. The deep learning-trained model was used to predict the soma regions (Fig. 1e) in the original grayscale image, achieving greater accuracy compared to the extraction results obtained using the *Hysteresis Thresholding* command. The *Image Gradient* command facilitated the delineation of boundaries between the adjacent MNs (Fig. 1f). Figure 1h and i demonstrated the application of the deep learning-trained model for predicting soma in 3D samples, with the red mask indicating the predicted soma contours. Additionally, the 3D reconstructed soma mask was shown in Fig. 1j. Figure S1 illustrated the detailed workflow for counting the number of MNs, while the signals extracted therein serve as seeds in the subsequent watershed segmentation procedure, enabling comprehensive and accurate segmentation. We next applied the *Marker-based Watershed inside Mask* command to segment individual MN. Each MN was labeled by unique random color, as shown in Fig. 1g. The parameters of the above commands were provided in Figure S2 for reference.

Besides, we proposed to validate the consistency of the pipeline by comparing the 2D cell diameters (Fig. S3a) and the 3D soma sizes (Fig. S3c). We extracted a region of approximately 100 µm thickness from a similar anatomical location in the lumbar spinal cord of each age group (only fully contained MNs in this region were measured). We then generated a Z-axis projection for this region and measured the 2D cell diameters. We also performed a 3D reconstruction of the MNs in that region (based on Deep learning command in Amira) and measured the 3D soma size. The results indicated that the frequency histograms of the 2D cell diameters (Fig. S3b) and the 3D soma sizes (Fig. S3d) are similar, though not completely identical. This is likely due to the limitations inherent in using 2D projections: the measurement of cell diameters is confined by the projection angle.

Overall, the generation of accurate soma masks is the most critical step in the whole semi-automated soma segmentation workflow. Traditional threshold segmentation struggles to accurately extract soma regions from the entire 3D image due to the high density of MNs in ChAT-eGFP mice and the complexity of dendritic branching. The deep learning module in Amira software significantly enhanced the accuracy and efficiency of soma boundary extraction. In addition, as the spinal cord was expanded by ~ 1.2 times in each dimension after clearing, the real soma size was 1.728 (1.2^3^) times smaller than the calculated soma size.

### Soma size changes of MMC and LMC neurons during postnatal development

MN subtypes are distinguished by their anatomical position, functional properties, connection specificity, and molecular profiles (Dasen 2022). The diversity of MNs is a functional necessity for the proper execution and control of movement during postnatal development. Mature MNs exhibit variations in soma size. It is not feasible to differentiate MNs based solely on soma size. However, the observed differences in soma size among MNs can be utilized as a supplementary indicator of differentiation. In previous studies, the soma size of MN was typically assessed by measuring the maximum cross-sectional area of MN in spinal cord slice. Changes in the 3D soma size and the spatial distribution of MN subtypes within the spinal cord during postnatal development remain poorly characterized.

**Fig. 2:**
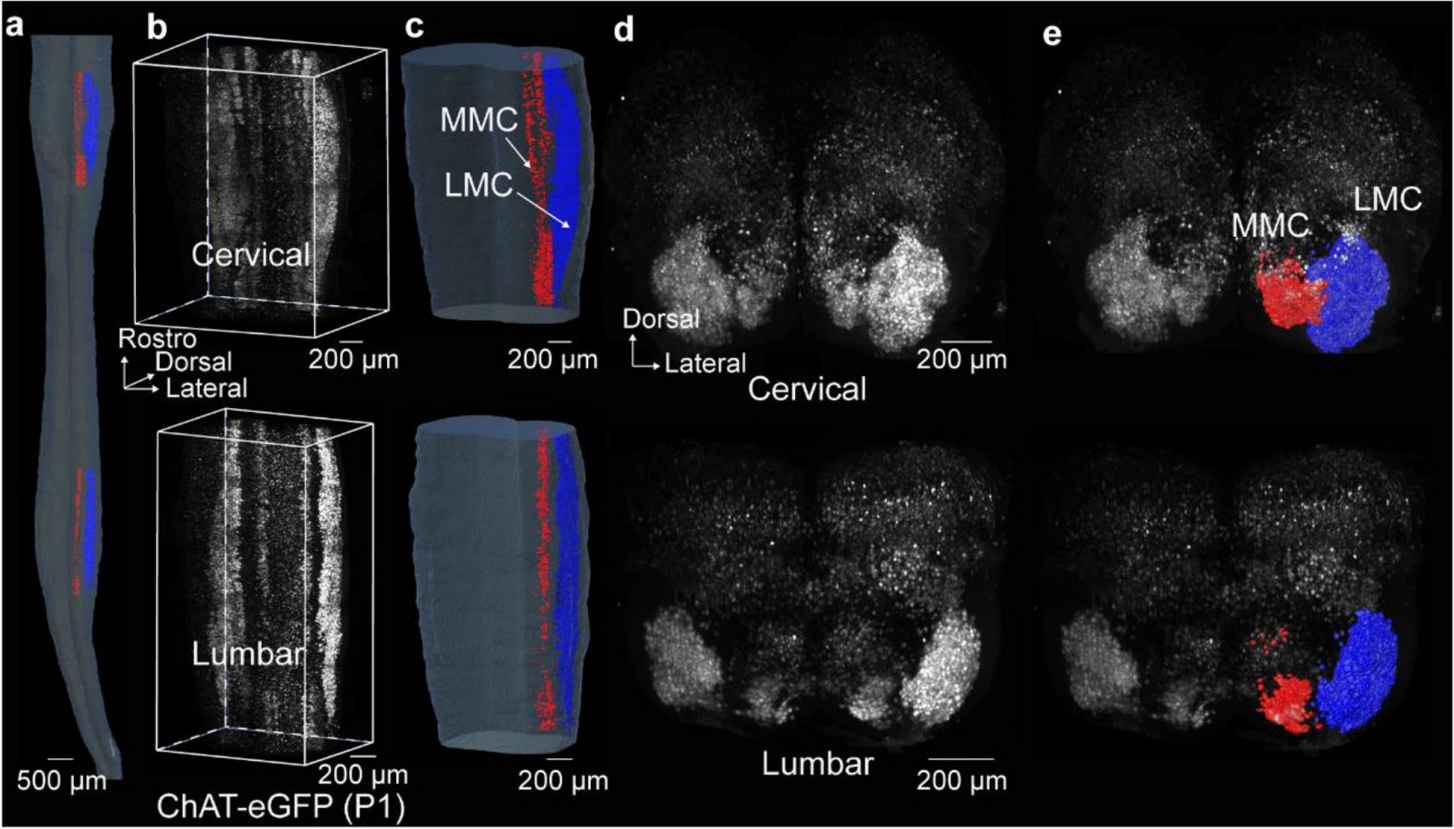
Reconstructed MMC and LMC neurons of P1 ChAT-eGFP. **a** The frontal view of the reconstructed MMC and LMC neurons of P1 ChAT-eGFP, with a scale bar of 500 μm. The red surface represented the reconstructed MMC, and the deep blue surface represented the reconstructed LMC. **b** Grayscale image of cervical cord (top), and the grayscale image of lumbar cord (bottom), with a scale bar of 200 μm. **c** Reconstructed LMC and MMC neurons of cervical cord (top), and lumbar cord (bottom), with a scale bar of 200 μm. **d** The transverse plane view of C5-T1 (top), and the transverse plane view of L1-L6 (bottom), with a scale bar of 200 μm. **e** The transverse plane view of C5-T1 (top), overlaid with the reconstructed MMC (depicted in red) and LMC (depicted in deep blue) neurons, with a scale bar of 200 μm. The transverse plane view of L1-L6 (bottom), overlaid with the reconstructed lumbar MMC (depicted in red) and LMC (depicted in deep blue), with a scale bar of 200 μm

We identified LMC neurons in the cervical (C5–T1) and lumbar (L1–L6) cord regions by correlating vertebral anatomy with spinal cord segments in mice (Fig. S4) (Stifani 2014; Dasen and Jessell 2009; Harrison et al. 2013). MMC neurons in these regions were also included in the analysis. The MMC and LMC columns were identified by their spatial locations within the spinal cord, and their distinct boundaries were clearly revealed through 3D image rendering (Video S1). Figure 2a-c and Video S2 display the cervical and lumbar spinal cord of a P1 ChAT-eGFP mouse, along with the reconstructed LMC and MMC neurons. The transverse planes of these two regions were presented in Fig. 2d. The top was the cervical region, and the bottom was the lumbar region. Figure 2e presented the transverse planes of the reconstructed LMC and MMC neurons.

**Fig. 3:**
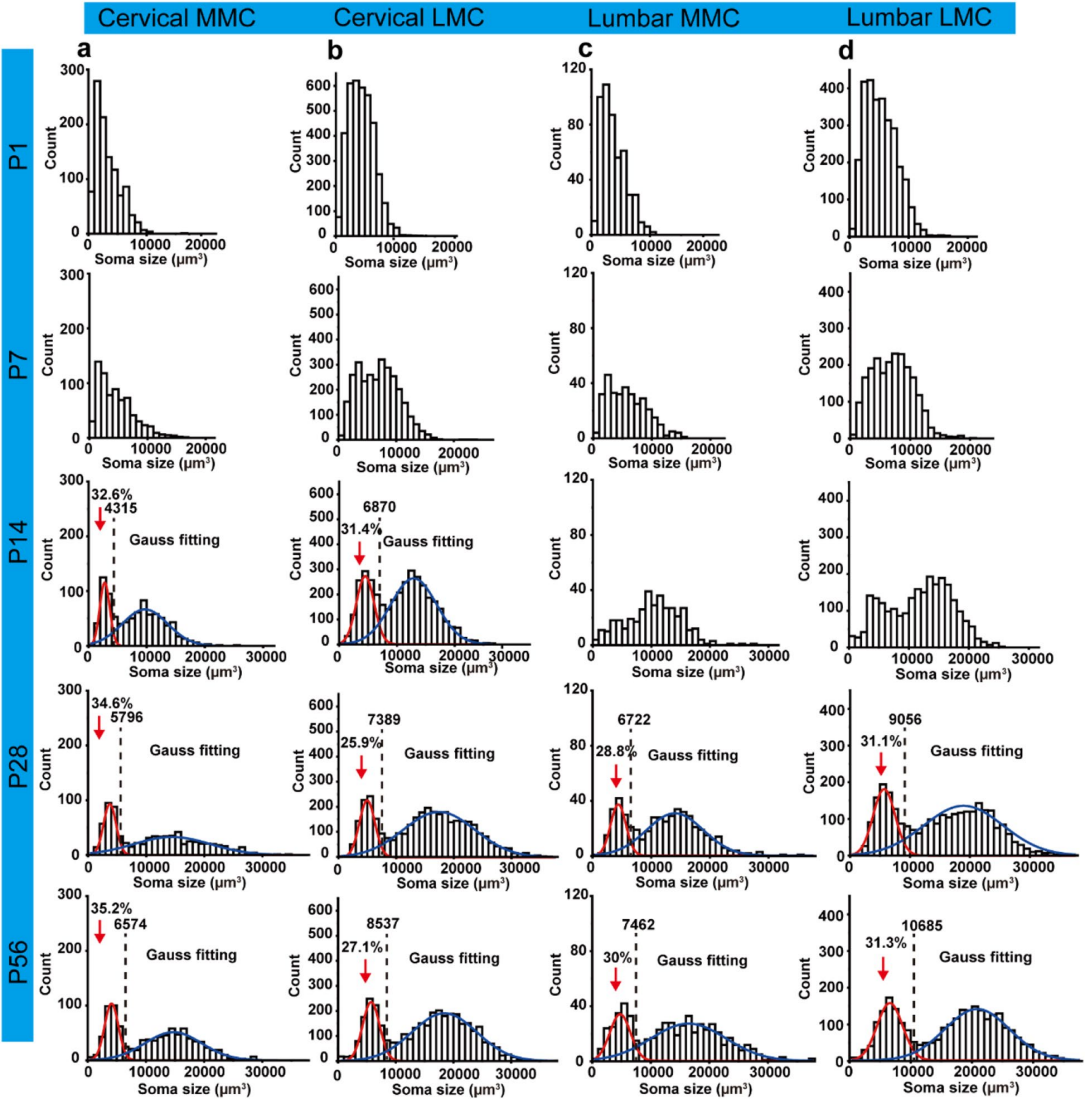
Histograms of the soma size distribution of MNs. **a** Histograms of the soma size distribution of MNs in the cervical MMC of P1, P7, P14, P28, and P56 ChAT-eGFP. **b** Histograms of the soma size distribution of MNs in the cervical LMC of P1, P7, P14, P28, and P56 ChAT-eGFP. **c** Histograms of the soma size distribution of MNs in the lumbar MMC of P1, P7, P14, P28, and P56 ChAT-eGFP. **d** Histograms of the soma size distribution of MNs in the lumbar LMC of P1, P7, P14, P28, and P56 ChAT-eGFP. The volume distribution histograms of MNs are analyzed using Gaussian fitting. The volume range is 0~38,000 µm^3^, with intervals of 1,000 µm^3^. *n* = 1 animal per group

The volumetric changes of MNs during postnatal development were characterized by quantifying the soma size of ChAT-eGFP mice at five ages (P1, P7, P14, P28, P56). A total of 1,049 (P1), 870 (P7), 889 (P14), 783 (P28), and 945 (P56) MNs were reconstructed in the cervical MMC. Figure 3a showed the histograms of soma size distributions in the cervical MMC of P1, P7, P14, P28, and P56 ChAT-eGFP mice. Similarly, 3,798 (P1), 3,688 (P7), 3,704 (P14), 3,516 (P28), and 3,553 (P56) MNs were reconstructed in the cervical LMC of ChAT-eGFP. Figure 3b demonstrated the histograms of soma size distributions in the cervical LMC of P1, P7, P14, P28, and P56 ChAT-eGFP mice. In the lumbar MMC, 498 (P1), 382 (P7), 375 (P14), 504 (P28), and 594 (P56) MNs were reconstructed. Figure 3c depicted the histograms of soma size distributions in the lumbar MMC of P1, P7, P14, P28, and P56 ChAT-eGFP mice. A total of 2,898 (P1), 2,482 (P7), 2,463 (P14), 2,762 (P28), and 2,750 (P56) MNs were reconstructed in the lumbar LMC. Figure 3d presented the histograms of soma size distributions in the lumbar LMC of P1, P7, P14, P28, and P56 ChAT-eGFP mice. The soma size (volume 3 d) of MNs in each motor column during postnatal development were presented in Table S2.

In the cervical MMC of P1 and P7 mice, the soma size distribution histogram exhibited a single peak. By P14, this distribution evolved to display two distinct peaks, corresponding to the differentiation of MNs into two subgroups. To analyze the soma size distribution histograms of the two MN subgroups, we applied the Gaussian fitting algorithm in Origin software to fit the histogram data for each subgroup. In the P14, P28 and P56 mice, MNs with smaller soma sizes accounted for 32.6%, 34.6% and 35.2%, respectively (Fig. 3a, indicated by red arrows). Similarly, in the cervical LMC of P1 and P7, the soma size distribution was represented by a single group. At P14, MNs with smaller soma sizes constituted 31.4% of the total population. In P28 and P56 mice, the proportion of smaller-sized MNs was 25.9% and 27.1%, respectively (Fig. 3b, indicated by red arrows). In the lumbar MMC of P1 to P14 mice, the soma size of MNs was distributed within a single group. In the mice at P28 and P56, MNs with smaller soma sizes presented 28.8% and 30% (Fig. 3c, indicated by red arrows), respectively. Additionally, the soma size of MNs in the lumbar LMC of P1 to P14 was represented by a single group. In the P28 and P56 mice, MNs of smaller soma size comprised 31.1% and 31.3% (Fig. 3d, indicated by red arrows), respectively.

### Spatial distribution of putative gamma and alpha MNs in the MMC and LMC

Friese et al. employed a combined approach of tissue sectioning and immunofluorescence staining techniques to analyze the maximum cross-sectional area of 800 MNs in the lumbar spinal cord of P21 wild-type mice (Friese et al. 2009). The researchers identified two distinct populations of MNs: Err3-positive and NeuN-negative MNs (widely recognized as the selective markers for γMNs) and Err3-negative and NeuN-positive MNs (widely recognized as αMNs). The researchers also discovered that Err3-positive and NeuN-negative MNs (putative γMNs) constitute 31% of the total population.

**Fig. 4:**
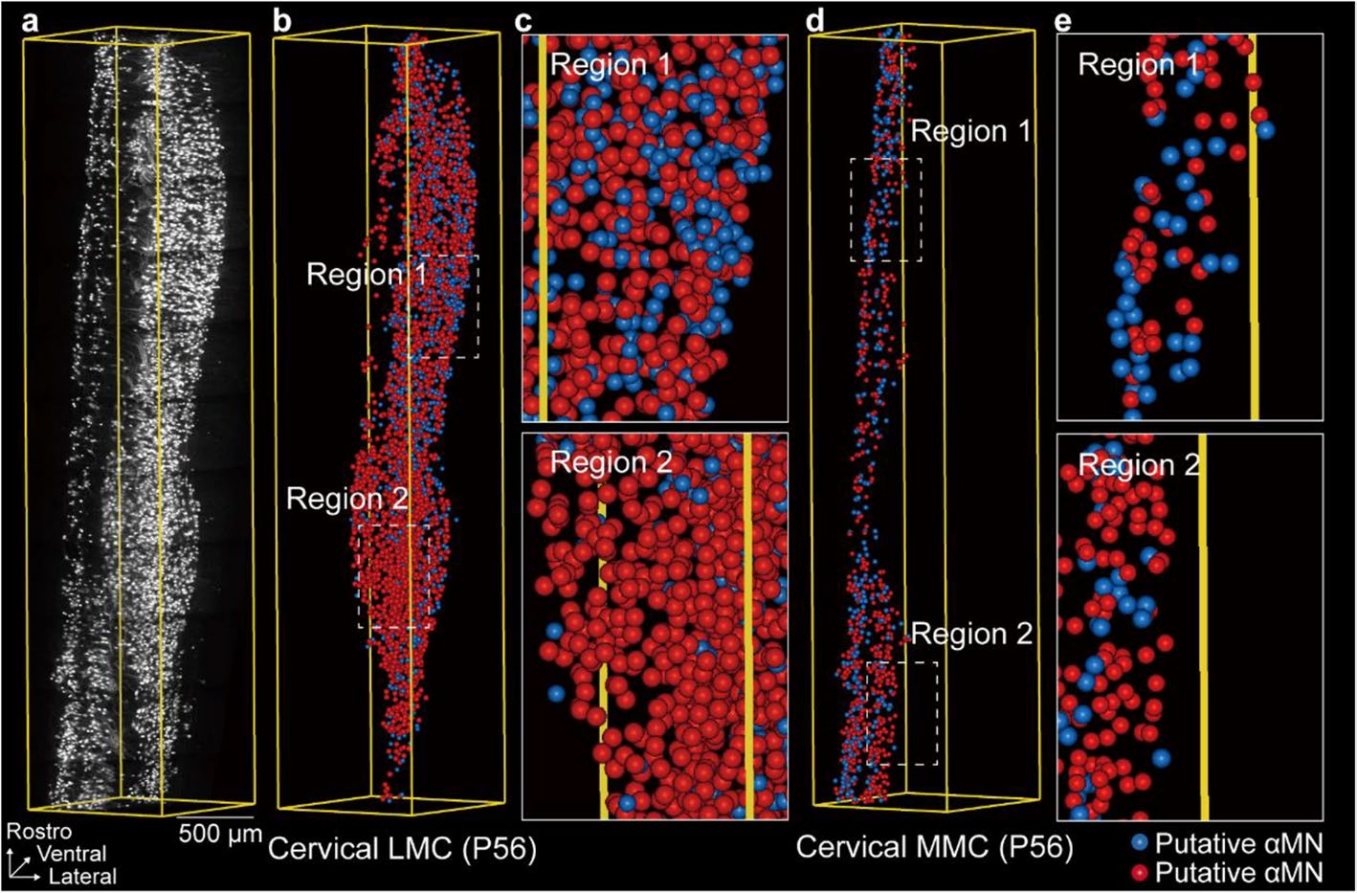
Spatial distribution pattern of putative γMNs and αMNs in the cervical cord. **a** Grayscale image of the cervical MNs of P56 ChAT-eGFP, with a scale bar of 500 μm. **b** Spatial distribution pattern of the putative γMNs and αMNs of the cervical LMC. **c** A magnified view of two regions in (**c**). **d** Spatial distribution pattern of the putative γMNs and αMNs of the cervical MMC. **e** A magnified view of two regions in (**d**)

Our observation was in accordance with the findings of Friese et al. We speculated that the subgroup of MNs with smaller soma sizes corresponds to γMNs, while the larger one corresponds to αMNs. We identified the spatial distribution patterns of putative γMNs and αMNs in the mature spinal cord using the results of Gaussian fitting. The x-coordinate of the intersection point between the two fitting curves defined the threshold that separates the putative γMNs (soma size smaller than the threshold) and αMNs (soma size larger than the threshold). In the cervical LMC of P56 ChAT-eGFP (Fig. 4a), putative γMNs are represented by blue spheres, and putative αMNs were represented by red spheres (based on Fig. 3b), as illustrated in Fig. 4b. These spheres were generated through 3D rendering using Amira software. Next, putative γMNs and αMNs in the cervical MMC of P56 ChAT-eGFP were also showed in blue spheres and red spheres (based on Fig. 3a), as illustrated in Fig. 4d. In the same way, the putative γMNs and αMNs in the LMC and MMC of the lumbar cord were represented as blue and red spheres, as shown in Figure S5. Through 3D rendering of the spatial distribution of putative γMNs and αMNs, we observed an interesting phenomenon: although smaller MNs (putative γMNs) account for approximately 30% of the total population, their spatial distribution was not uniformly proportional to this percentage (Fig. 4c, e). While the spatial distribution pattern of putative γMNs and αMNs was preliminarily predicted through soma reconstruction and 3D rendering, confirming these observations with immunohistochemical analyses (e.g., detecting Err3 and NeuN) remains a key step for future exploration.

### Dendrites extraction with Amira

In order to investigate the dendritic arborization patterns of MNs at the single-cell level during development, we retrogradely labeled the MNs innervating the tibialis anterior muscle (TA, flexor) and the gastrocnemius lateralis muscle (GL, extensor) with AdV at P4, P14, and P56 (Figure S6a–f). Bellingham et al. performed quantitative analyses of dendritic morphology in retrogradely or individually dye-filled MNs in age groups spanning approximately three to four days postnatally (Fogarty et al. 2016). In addition to these previously established time points, our choice of P4, P14, and P56 was also guided by several biological considerations. For P4, we performed viral injections on P1 rather than immediately after birth because very young pups (P0) are particularly fragile and have a high risk of postoperative mortality. Given that the AdV expression window is around 72 hours, P4 represents a practical and feasible starting point for our measurements. P14 was selected because the third postnatal week (spanning P14 to P21) is marked by significant transcriptional and functional maturation of motor circuits, accompanied by notable behavioral changes, making it a critical period for developmental studies (Patel et al. 2022). Finally, P56 corresponds to the age at which mice reach sexual maturity, thus enabling the investigation of the dendritic architecture of mature MNs (Walker et al. 2009).

Next, multiple site injections of AdV-eGFP and AdV-tdTomato were administered along the three-dimensional distribution of each target muscle’s motor endplate (Xu et al. 2021). A mixture of AdV and 1% Fast Green was used to aid in visualization (Fig. S6g, top). After 72 hours, the spinal cord was dissected and the fluorescent signals in the target muscles were observed under a fluorescence stereomicroscope. The dissected spinal cord then underwent tissue clearing and was imaged using TLSM (bottom of Fig. S6g). We employed a quantitative analysis of the spatial distribution of MNs innervating TA and GL muscles at P4, P14, and P56. As age increased, MNs were located progressively farther from the lateral and ventral borders of the spinal cord (Fig. S6h–j).

**Fig. 5:**
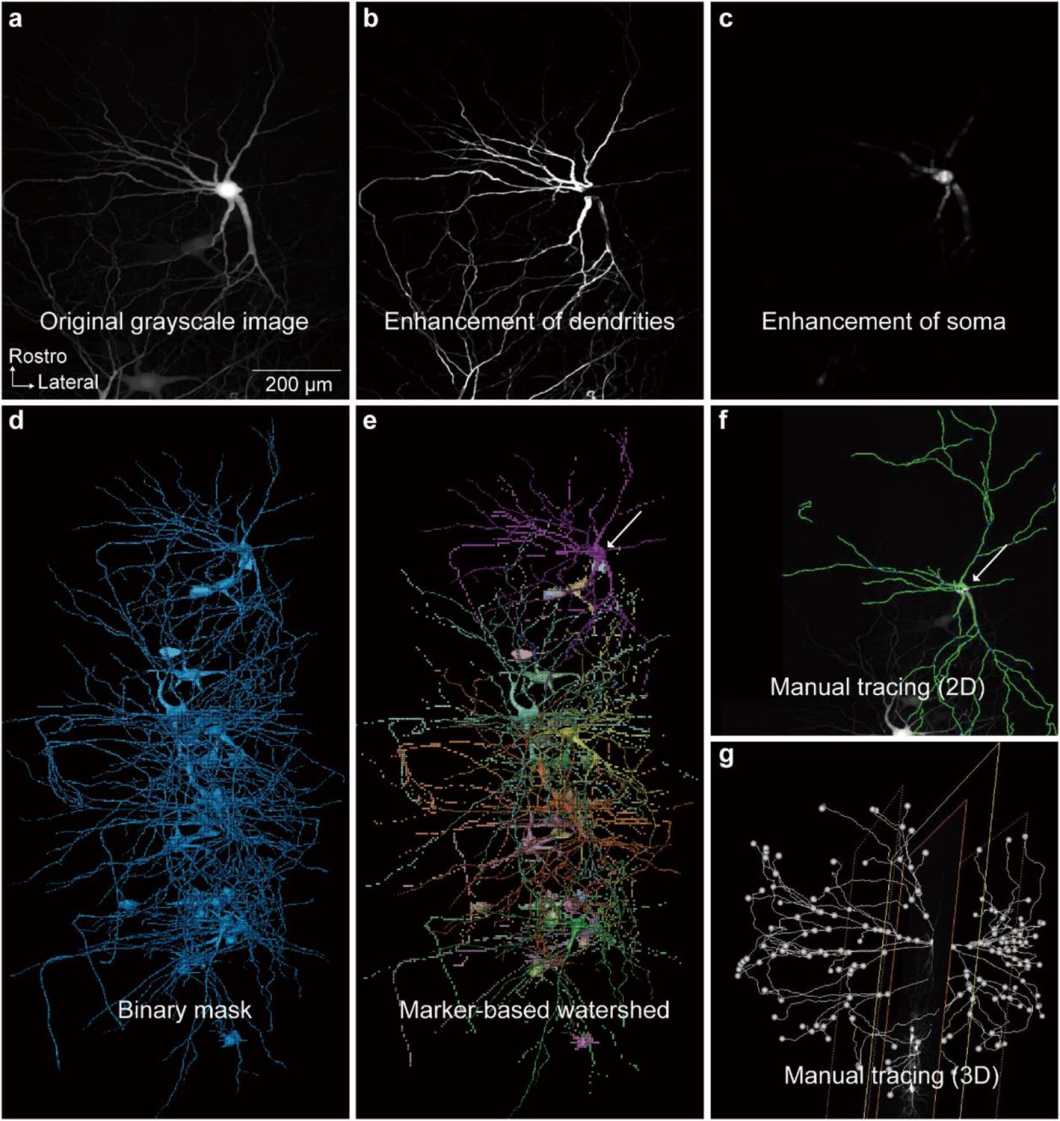
The semi-automatic method for dendritic tree extraction. **a** Grayscale image of P14 TA MNs retrogradely labeled by AdV, with a scalebar of 200 μm. **b** Dendritic structure was enhanced by *Structure Enhancement Filter (Rod Model)* command. **c** Somatic structure was enhanced by the *Structure Enhancement Filter (Ball Model)* command. **d** The *Interactive Thresholding* command was employed to transform grayscale images into binary image. **e** The *Marker-based Watershed inside Mask* command assigned distinct labels to each MN. **f** The *Centerline Tree* command generated the dendritic skeleton. **g** Manual tracing and editing were performed with the *Filament Editor*

A semi-automatic method for dendritic tree extraction was developed using Amira (Video S3). This method involved reconstructing dendritic arborization and quantifying the number and length of dendrites. First, the *Structure Enhancement Filter (Rod Model)* command was used to highlight tubular structures (Fig. 5a, b), while the *Structure Enhancement Filter (Ball Model)* command was applied to enhance spherical structures (Fig. 5c). Next, the *Interactive Thresholding* command converted the grayscale images into binary images, visualized as blue masks (Fig. 5d). Subsequently, the *Marker-based Watershed inside Mask* command assigned distinct labels to each MN, with each label displayed in a different color (Fig. 5e). The *Centerline Tree* command then extracted the centerline of the segmented MNs, producing the dendritic branch skeleton (Fig. 5f). Finally, manual tracing and editing were performed to ensure the complete and accurate extraction of the distal dendrites (Fig. 5g).

### Dendritic branching patterns of MNs that innervate the flexor and extensor muscles at P4, P14 and P56

**Fig. 6:**
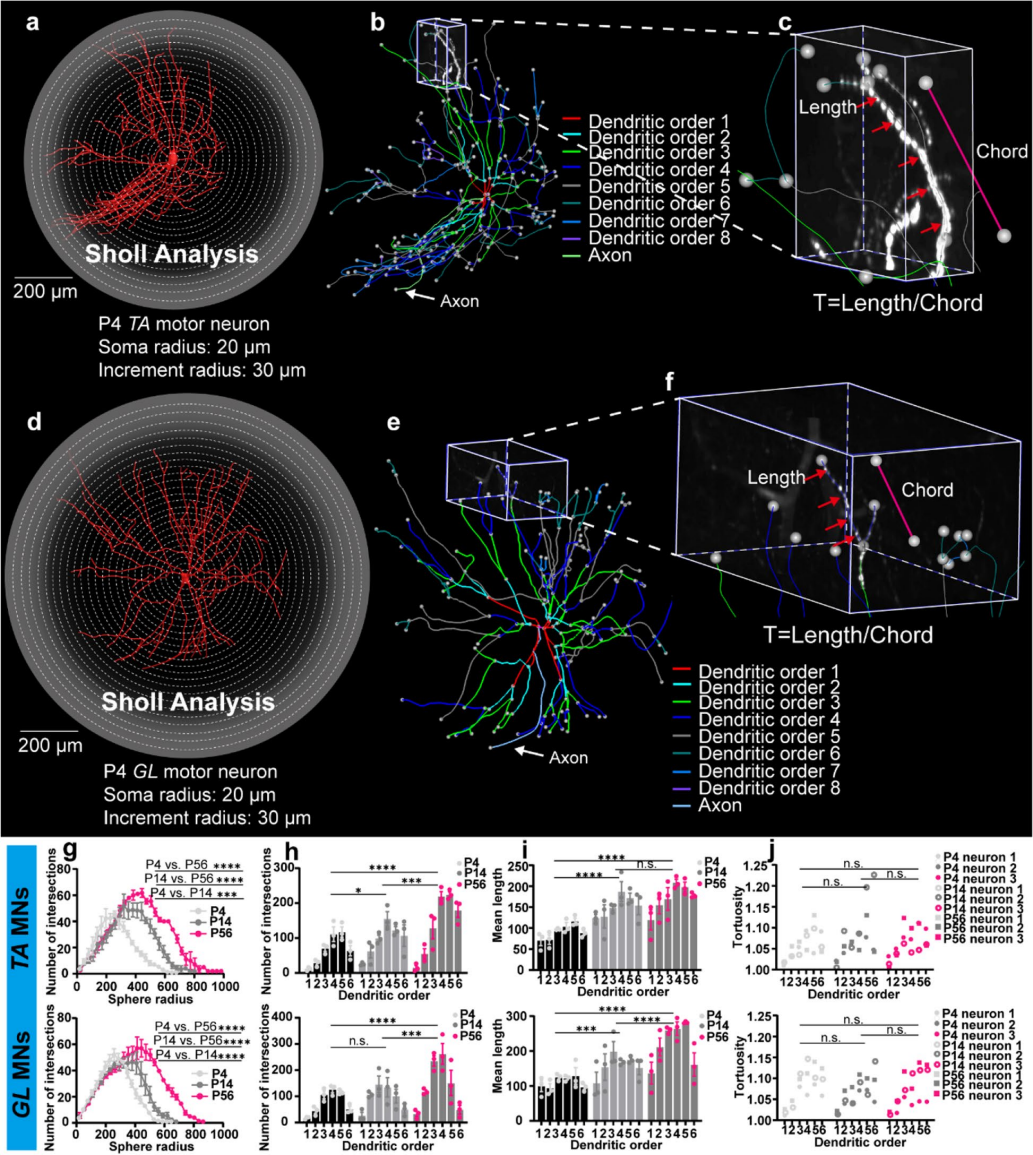
Quantitative analysis of the dendritic structures of the TA and GL MNs at P4, P14 and P56. **a** 3D surface reconstruction of a single TA MN, obtained using the Amira software, with a scalebar of 200 μm. **b** Skeletonize of a single TA MN, with dendritic branches color-coded by branch order. **c** Tortuosity of a single dendritic branch of a TA MN. The magenta line represents the chord length, and the red arrows indicate the 3D length of the dendritic branch. **d** 3D surface reconstruction of a single GL MN, obtained using the Amira software, with a scalebar of 200 μm. **e** Skeletonize of a single GL MN, with dendritic branches color-coded by branch order. **f** Schematization of tortuosity of a single dendritic branch of a GL MN. **g** Quantitative comparison for Sholl analysis of TA (top) and GL (bottom) MNs at P4, P14, and P56. **h** The number of crossings of each branch of TA (top) and GL (bottom) MNs with concentric circles for each branch order at P4, P14 and P56. **i** The mean length of the first six dendritic orders of TA (top) and GL (bottom) MNs at P4, P14, and P56. **j** The tortuosity of the first six dendritic orders of TA (top) and GL (bottom) MNs at P4, P14, and P56. All data are presented as mean ± SEM. *p*-values were obtained from two-way ANOVA with Tukey’s multiple comparisons test. Significance levels are indicated as follows: *p < 0.05, **p < 0.01, ***p < 0.001, ****p < 0.0001. Error bars represent standard error of mean. *n* = 3 neurons, from two animals

We reconstructed the 3D skeleton of dendrites of 3 motor neurons from the TA (Fig. S7a) and GL (Fig. S7b) motor pools at P4, P14 and P56. The reconstructed neural surface included the soma, dendrites, and axon (Fig. 6a, d), with dendritic branches color-coded by branch order using unique random colors for each order (Fig. 6b, e). To evaluate changes in dendritic complexity during development, we conducted a Sholl analysis (Fig. 6a, d) (Sholl 1953), which revealed that the number of intersections between the dendrites and concentric circles initially increased and then decreased (Fig. 6g). By integrating the Sholl analysis results with dendritic order information, we found that the region of highest dendritic density was associated with the 4 th or 5 th dendritic order (Fig. 6h). We next quantified the mean length of the first six dendritic orders in both TA and GL MNs at each developmental stage (Fig. 6i). In TA MNs, the mean length of each dendritic branch increased from P4 to P14, with no statistical difference detected between P14 and P56 (Fig. 6i, top). In contrast, GL MNs displayed a continuous increase in the mean length of each dendritic branch from P4 to P56 (Fig. 6i, bottom). In addition, it has been demonstrated that dendrites increase their probability of connecting with target synapses by increasing their curvature (Stepanyants et al. 2004; Rothnie et al. 2006). We also measured tortuosity-defined as the ratio of curve length to chord length (Fig. 6c, f) in the first six dendritic orders of TA and GL MNs at P4, P14, and P56. We compared the P1 vs. P14 vs. P56 groups. No statistical differences in tortuosity were observed during development (Fig. 6j).

## Discussion

In this study, we started with utilizing a combination of TLSM and CUBIC-L to visualize the MNs of ChAT-eGFP mice during postnatal development. The imaging and tissue clearing techniques provided rapid acquisition of large-volume samples with submicron resolution and obviate the need for physically sectioning. Then, we developed a protocol to effectively segment soma from large-volume and high-throughput samples using Amira. Commercial software typically provides standardized processing procedures, which enhance the reproducibility.

Image post-processing generally involves cell counting and 3D reconstruction. For example, in the research by McDevitt et al., the spot assay in Imaris was used to count V2a interneurons (McCreedy et al. 2021). Differences in the soma sizes of V2a interneurons were observed, but quantitative analysis was not performed. Furthermore, Chen et al. employed a machine learning–based method within Imaris to count and reconstruct the Vglut2+ and Vgat+ interneurons in mice at 2-, 14-, and 35-days post–spinal cord injury, within a 3-mm segment of the thoracic spinal cord (T7–T10) (Li et al. 2024). Chen et al. conducted extensive and rigorous statistical analyses on both the cell numbers and soma sizes (Li et al. 2024). Their study revealed that neurons with volumes of less than 1000 µm^3^ were predominantly distributed in the dorsal horn, whereas neurons with volumes exceeding 8000 µm^3^ were more concentrated in the ventral horn. In the present study, we focused on MNs located in the ventral horn. Notably, α-MNs, which account for approximately two-thirds of the total MN population, are the largest neurons in the spinal cord. Regarding the soma sizes of the lumbar LMC (Fig. S8a-e), the median volumes were 5039 µm^3^ (P1), 7031 µm^3^ (P7), 12400 µm^3^ (P14), 15312 µm^3^ (P28) and 17781 µm^3^ (P56). Although the measurement approach differs from that of Chen et al., the MN soma sizes measured in the present study remain reasonable. Additionally, Bellingham et al. measured the mean dendritic length of hypoglossal MNs in 300 µm-thick sections (Kanjhan et al. 2016a). The mean dendritic length obtained in the present study is on the same order of magnitude as that reported by Bellingham et al, further validating the accuracy of the dendritic reconstruction method in the present study.

In terms of AI-based algorithms, Chen et al. integrated Labkit with Imaris software to label and segment neurons (Li et al. 2024). To train Labkit for neuron recognition in 3D images, approximately 10 cells of varied shapes and sizes were manually selected per horizontal single-plane view—amounting to roughly 150 cells for each spinal cord. In the present study, due to the complex neuronal morphology and minimal intercellular spacing of MNs, non-AI-based commands in Amira failed to accurately recognize the somas. As a result, we employed Amira’s built-in deep learning command (DL Training–Segmentation 3D command) to solve the problem. Approximately 30 MB of data per spinal cord (corresponding to about 150~200 MNs) was extracted following the semi-automated workflow depicted in Fig. 1 to train the deep learning model. The trained model was subsequently applied to the corresponding spinal cord, enabling accurate segmentation.

The configuration of training parameters for the deep learning command used in this study—which ultimately determines prediction accuracy—is heavily dependent on the GPU performance of the workstation. Based on our workstation’s hardware setup (Fig. S8f), the upper limit for these training parameters is illustrated in Figure S2c. In other words, on a high-performance GPU platform, the algorithm can optimize training parameters to enhance predictive performance (Gibbs et al. 2021). However, it’s accompanied by a limitation: when hardware resources are constrained, it becomes challenging to optimize the training parameters, which may reduce prediction accuracy. Overall, the AI-based algorithm used in this study necessitates a trade-off between hardware investment and prediction performance.

The 3D reconstruction of MNs across multiple spinal cord segments was performed in this study, effectively minimizing the statistical bias introduced by variations in sectioning direction inherent to 2D methods. Additionally, the number of MNs reconstructed from one mouse (for example, 3,798 MNs were reconstructed in the P1 cervical LMC) exceeded the total counts reported in previous studies that used two-dimensional slices from five mice. We acknowledge that utilizing a single sample (*n*= 1) has accidentality. Therefore, the focus of the present study is on establishing a pipeline for the 3D reconstruction of MNs and providing descriptive observations of postnatal changes in soma size.

MNs undergo a differentiation process during development, resulting in the emergence of three subtypes: αMNs, βMNs and γMNs (Manuel and Zytnicki 2011). This regulation is crucial for the maintenance of muscle tone, the adjustment of muscle reflexes, and fine motor control. Previous studies have employed immunofluorescence and tissue sectioning techniques to analyze the cross-sectional area of αMNs and γMNs. The histogram of the cross-sectional area of the soma revealed the presence of two distinct groups. The smaller group was identified as putative γMNs, constituting approximately 30% of the total (Friese et al. 2009; Kang et al. 2024a; Shneider et al. 2009). Although smaller MNs (putative γMNs) account for approximately 30% of the total population, the distribution of putative αMNs and γMNs differs regionally. In certain areas, putative αMNs predominate, whereas in others, putative γMNs are more prevalent. Although further validation was not conducted, this observation suggests that the differences in αMN and γMN distribution may depend on the specific muscle type they innervate. For example, some motor pools may contain more αMNs to recruit a larger number of extrafusal muscle fibers. Consequently, the corresponding spinal cord region would exhibit a concentrated presence of αMNs. Conversely, other motor pools might harbor a higher proportion of γMNs to maintain muscle spindle sensitivity, resulting in a region enriched for γMNs. To verify this hypothesis, a feasible approach is proposed. First, retrograde tracers—such as Fluoro-Gold, Fluoro-Ruby, or Cholera Toxin Subunit B-Alexa Fluor 488—can be injected into the target muscle to label the motor pool that controls it (Han et al. 2019; Mantilla et al. 2009). Next, by combining this technique with immunofluorescence staining using markers specific for αMNs and γMNs, the proportions and spatial distributions of these neuronal subtypes within the motor pool can be confirmed. In terms of disease relevance, amyotrophic lateral sclerosis (ALS) selectively affects the larger αMNs that are characterized by complex synaptic connections and high metabolic demand (Lalancette-Hebert et al. 2016; Nijssen et al. 2017). Comparing the ratios and spatial distributions of αMNs and γMNs in ALS model mice and wild-type mice might provide potential strategies for drug intervention. The present study also revealed that MNs in the cervical and lumbar regions differentiated into two groups with distinct soma sizes around P14, which was consistent with the results of the aforementioned studies. We speculated that the alterations in MN soma size during development may serve as an indicator of differentiation.

Previous studies have indicated that the muscle groups of the forelimbs develop slightly earlier than those of the hindlimbs during the initial few days after birth (Zhu and Tabin 2023; Martin 1990). For instance, at P11, the phosphorylation levels of specific proteins, such as Pak1 and Pak2, exhibit disparities between the forelimb and hindlimb muscles (Joseph et al. 2019). This early developmental advantage endows the forelimbs with greater robustness when neonatal mice commence movement and exploration of their surrounding environment. The present study analyzed the 3D soma size of MNs in the cervical region and revealed that MNs innervating the forelimbs differentiate into two distinct groups with varying soma sizes by P14. However, the differentiation of MNs in the lumbar region was delayed until after P14. This observation indicated that the maturation and differentiation of MNs innervating the hindlimbs occur at a later stage than those innervating the forelimbs.

Additionally, our findings revealed that the distribution of putative γMNs and αMNs is not uniform. In certain regions, putative γMNs are more abundant, while in others, putative αMNs are more prevalent. Although no further validation was performed, this finding suggested that the proportion of γMNs and αMNs may vary depending on the muscle fibers they innervate. It’s practicable to inject retrograde tracers into the target muscle, which could allow for a predictive analysis of the proportion of γMNs and αMNs by examining the soma size. We speculated that the function and influence on the ALS-like pathologies of the particular muscle could be further inferred from the proportion of γMNs and αMNs in the corresponding motor pool. This approach may also provide a theoretical basis for understanding how the motor system efficiently synchronizes various motor pools and for developing more effective exercise strategies.

It was observed that MNs retrogradely labeled with AdV exhibited robust fluorescent expression, while the distribution of fluorescence signals was uneven. In particular, the fluorescence intensity in the soma region differed by a factor of 2~3 compared to the apical dendrite region. To address this problem, we employed extensive manual tracing to extract the complete dendritic arborization. The analysis demonstrated that the number of dendritic branches of MNs, regardless of whether they innervate flexors or extensors, tended to increase and then decrease. This indicates that the number of branches is less near the soma and proximal dendrites, with the majority of branches situated in the mid-dendritic region. It is noteworthy that the length of dendrites of MNs increases with age, and the pattern of dendrites constructed by P4 is similar to that of mature adults. The intricate structure of dendrites is primarily a consequence of their function in integrating synaptic inputs while simultaneously minimizing metabolic costs. Furthermore, comprehensive understanding the dendritic structures of MNs can also facilitate the development of efficient mathematical models of neural networks for motor control.

In summary, this study provided a method for capturing the 3D morphology of MNs during postnatal development by combining TLSM imaging system, CUBIC-L tissue clearing protocol, and AdV labeling. Moreover, this study established highly reproducible image analysis methods to quantitatively analyze the morphological changes of MNs during postnatal development in two aspects: differentiation of soma size and dendritic architecture. The findings provide new insights into the maturation, differentiation, and functional properties of MNs, allowing a deeper understanding of the motor system during development.

### Limitations

The achievement of high-resolution imaging of MNs in the whole spinal cord requires the handling of tens to hundreds of terabytes of data. Despite the establishment of a semi-automatic soma segmentation method based on deep learning, the computational process remains time-consuming and requires high-performance workstations. Consequently, in the initial stages of our investigation, the changes in soma size were restricted to the cervical and lumbar LMC and MMC of a single mouse per age group. This limitation may affect the generalizability of our findings, as the data from a single mouse may not fully represent the variability and complexity observed in a broader population. Besides, how can γMNs be differentiated before the clear bifurcation of their soma size into two distinct groups? This question cannot be answered on the basis of morphological changes alone. Further explorations involving specific molecular markers and electrophysiological properties of αMNs, βMNs and γMNs are required. Over the past decade, numerous molecular markers for γMNs have been identified (Zuccaro et al. 2021; Kang et al. 2024b). However, it should be noted that many of these markers can be expressed in both αMNs and βMNs. Combing multiple molecular markers is essential.

In examining the dendritic arborization of MNs, the TA and GL, which are superficial muscles, were labeled. The present study did not examine the dendritic branching patterns of MNs that innervate deeper muscles. This introduces a limitation, as the structural and functional characteristics of MNs may differ between the superficial and deep muscles. Furthermore, the intricate and compact structure of MN distal dendrites, necessitated substantial manual calibration to ensure accurate segmentation. The labor-intensive nature of this process resulted in a reduction in the overall throughput of data analysis, thereby limiting the number of MNs that could be selected for dendrite analysis to only three per age group.

Furthermore, the dendrite tracing approach only extracted the centerline of the dendrites. The centerline extraction algorithm of Amira identifies the highest intensity path within a dendrite, rather than capturing the full grayscale signal present in the original image. As a result, this method does not yield information regarding the diameter, surface area, and volume of a dendrite. The diameter of a dendrite has a direct effect on its electrical conductivity. Measuring the diameter of a dendrite requires the application of additional analysis algorithms.

## Methods

### Animal preparation

The collection of mice tissues used in this study was approved by the Institutional Animal Care and Use Committee of the Westlake University (Approval No: 19–035-GL). All mouse lines were kept on C57BL/6 J background and were housed in a standard 12:12 light-dark cycle.

C57BL/6 J was purchased from Shanghai Jihui Laboratory Animal Care Co., Ltd..ChAT-eGFP (B6.Cg-Tg(RP23 - 268L19-EGFP)2Mik/J, Stock No: 007902) was kindly provided by Dr. Liang Wang from Zhejiang University.

### Tracer preparation

An expression cassette comprising the cytomegalovirus promoter and the cDNA encoding enhanced green fluorescent protein (eGFP) packaged into adenovirus serotype 5 was purchased from OBiO Technology (Shanghai) Corp., Ltd.. The viral titer was 1.58 × 10^11^ pfu/ml. The solutions were stored at − 80℃ until use.

### Intramuscular injections

#### Injection time

Adenoviruses enter cells through receptor-mediated endocytosis by binding with high affinity to receptors such as coxsackie and adenovirus receptors (CAR) and members of the integrin receptor family. In skeletal muscle, CARs are primarily confined to the neuromuscular junction (also referred to as the motor endplate, MEP) (Andrew Paul Tosolini and Morris 2016). After internalization, adenoviral particles are transported by cytoplasmic dynein along the MN axon’s microtubule network to the soma (Arnberg 2012). Therefore, CAR expression and the transportation distance along the microtubules are key factors governing gene delivery efficiency. With increasing age, the expression of CARs in skeletal muscle decreases (Sinnreich et al. 2005). As a result, the total number of transduced MNs following intramuscular injections of Ad.eGFP is higher in juvenile mice than in adult mice. In addition, the longer axon lengths observed in older animals result in increased transportation distances for AdV particles. Thus, the age of the animals directly affects vector spread and transport efficiency. Based on these considerations, the period post-delivery for optimal adenoviral particle uptake was estimated to be 3 days in juvenile mice and 5 days in adult mice. Accordingly, to label the dendritic morphology of MNs in P4 and P14 mice, intramuscular injections were performed at P1 and P14, respectively. For labeling motor neurons in P56 mice, the injection was carried out at P51.

#### Injection site and depth

The location of injection sites based on the 3D distributions of MEP in the tibialis anterior and gastrocnemius lateralis, according to the published literatures (Xu et al. 2021; A. P. Tosolini and Morris 2016). α-bungarotoxin was used to label all MEPs in the gastrocnemius and tibialis anterior muscles. Three-dimensional maps for intramuscular injection of dyes or tracers were created. The injection parameters in the present study were set according to the methods described by these researches. The mice were deeply anaesthetized, and the right hind limb was secured with medical tape, shaving the tibialis anterior and gastrocnemius lateralis muscle regions. In the mice at P1, three sites were injected with an injection depth of 0.1~0.2 mm; In the mice at P11, five sites were injected with an injection depth of 0.3~0.6 mm; In the mice at P51, contained seven injection sites with an injection depth of 0.6~1.2 mm. Each site was injected with 0.5 μl of AdV.

### Spinal cord harvest

After the intramuscular injections, the mice were kept for 3 days to allow for optimal retrograde transport of neuronal tracer. Then, mice were deeply anaesthetized and perfused with 37 ℃ saline followed by 4 ℃ 4% paraformaldehyde. The mouse spinal cord was dissected following a previously reported protocol (Kennedy et al. 2011). The dissected spinal cord was loosely wrapped with a 200 μm Nylon mesh and bound to a perforated Teflon plate with surgical sutures so that the spinal cord can maintain the straight shape through the sample preparation process. The bound spinal cord was placed in 4% PFA overnight at 4 °C with gentle shaking.

### Preparation of cleared and expanded mouse spinal cord samples

The preparation of cleared spinal cord samples was referenced from CUBIC-L protocol (Matsumoto et al. 2019; Tainaka et al. 2018). Preparation of the cleared spinal cord: First, the fixed spinal cord was immersed in the delipidation solution (10 wt% N-butyldiethanolamine, 10 wt% Triton X- 100 and 80 wt% dH2O) at 37 °C for three days with gentle shaking. The delipidation solution was replaced every 24 hours. Next, the delipidated spinal cord was RI matched by immersing the spinal cord in the RI matching solution of RI ~ 1.49 (25 wt% urea, 22.5 wt% sucrose, 22.5 wt% antipyrine, 10 wt% triethanolamine) at 25 °C for two days with gentle shaking. The RI matching solution was replaced every 24 hours. Finally, the RI matched spinal cord was embedded in 2% agarose gel made with the RI matching solution. The cleared and embedded spinal cord was mounted on the sample holder by attaching the sample to the magnets of the sample holder and imaged with the imaging system using Silicone oil as the imaging buffer (Feng et al. 2022).

### Microscopy

The configuration of our imaging system was described in our previous publications (Chen et al. 2020). It is capable of imaging centimeter-scale cleared tissues with micron-scale to submicron-scale spatial resolution by using real-time optimized tiling light sheets and detective objectives with various numerical apertures (Olympus XLSLPLN10XSVMP, Nikon Plan Apo 10x Glyc, or Nikon CFI90 20XC Glyc). The resolving ability can be improved to ~ 70 nm in combined with tissue expansion. The microscope conducts multicolor imaging sequentially for up to 5 colors with the excitation wavelengths of 405 nm, 488 nm, 515 nm, 561 nm, and 638 nm.

### Image analysis

Image processing, registration and merging protocols were described in our previous publications (Feng et al. 2021). Soma segmentation and dendrite tracing were conducted using Amira semiautomatically, and the methodologies are described in detail in Fig. 1 (soma segmentation) and Fig. 5 (dendrite tracing).

## Supplementary Information

Supplementary Material 1: Figure S1. The cell counting method. Figure S2. Parameters of the commands used in the soma segmentation process. Figure S3. The cervical (C5-T1) and lumbar (L1-L6) cord of P56 ChAT-eGFP. Figure S4. Spatial distribution pattern of putative γMNs and αMNs in the lumbar cord. Figure S5. Spatial distribution of MNs innervating TA and GL muscles during development. Figure S6. The skeletonized dendrites of TA and GL MNs. Figure S7. Frequency histograms of 2D cell diameter and 3D soma size of MNs during development. Figure S8. Soma size of MNs during postnatal development at the 25% percentile, median, and 75% percentile.

Supplementary Material 2: Table S1. The parameter settings of the deep learning command for soma segmentation. Table S2. The soma size (volume3d) of MNs in each motor column of cervical and lumbar cord at P1, P7, P14, P28 and P56.

Supplementary Material 3: Video S1. The 3D image rendering of the MMC and LMC columns with a clear boundary of P56 ChAT-eGFP. Related to Figure 1. Video S2. The 3D image rendering of the cervical cord with the segmented MNs and the reconstructed LMC and MMC of P1 ChAT-eGFP. Related to Figure 1. Video S3. The 3D image rendering of the sparsely labeled MNs using AdV, the automatically segmented MNs as well as the manually traced distal dendrites. Related to Figure 5.

## Data Availability

The datasets used and/or analyzed during the current study are available from the corresponding author on reasonable request.

## Abbreviations

3D: Three-dimensional
AdV: Adenovirus
ALS: Amyotrophic lateral sclerosis
C: Cervical
CAR: Coxsackie and adenovirus receptors
CNS: Central nervous system
eGFP: Enhanced green fluorescent protein
GL: Gastrocnemius lateralis
L: Lumbar
LMC: Lateral motor column
MEP: Motor endplate
MMC: Median motor column
MN: Motoneuron
P: Postnatal day
RI: Refractive index
SpMN: Spinal motoneuron
T: Thoracic
TA: Tibialis anterior
TLSM: Tiling light sheet microscope
