# Supplementary material for: Morphological segmentation with tiling light sheet microscopy to quantitatively analyze the three-dimensional structures of spinal motoneurons
Source: Cell Regen. 2025 May 14;14:17. doi: 10.1186/s13619-025-00231-3 (PMC12075063; doi:10.1186/s13619-025-00231-3)
Supplement: Supplementary file 1 — Supplementary Material 1: Figure S1. The cell counting method. Figure S2. Parameters of the commands used in the soma segmentation process. Figure S3. The cervical (C5-T1) and lumbar (L1-L6) cord of P56 ChAT-eGFP. Figure S4. Spatial distribution pattern of putative γMNs and αMNs in the lumbar cord. Figure S5. Spatial distribution of MNs innervating TA and GL muscles during development. Figure S6. The skeletonized dendrites of TA and GL MNs. Figure S7. Frequency histograms of 2D cell diameter and 3D soma size of MNs during development. Figure S8. Soma size of MNs during postnatal development at the 25% percentile, median, and 75% percentile. [file 13619_2025_231_MOESM1_ESM.docx]

# Supplemental Figures


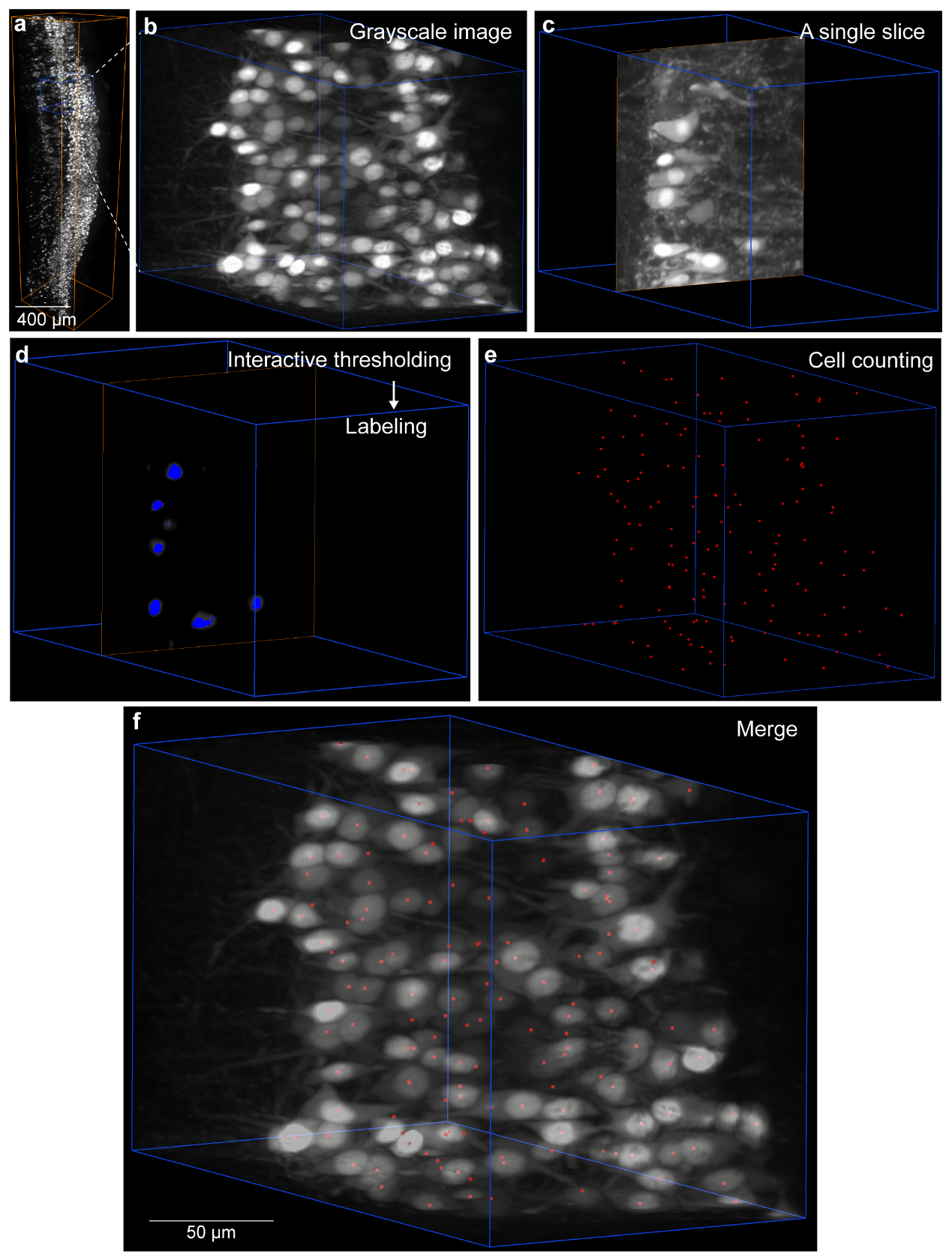


Figure S1. The cell counting method. (a) The 3D rendering of the lumbar MNs of P56 ChAT-eGFP, with a scale bar of 400 μm. (b) A magnified region from (a). (c) A single slice form (b). (d) Using the *Interactive Thresholding* command to binarize the grayscale image. (e) Counting the cell number of MNs (red dots) with the *Labeling* command. (f) Cell counting merged with the 3D grayscale image, with a scale bar of 50 μm.


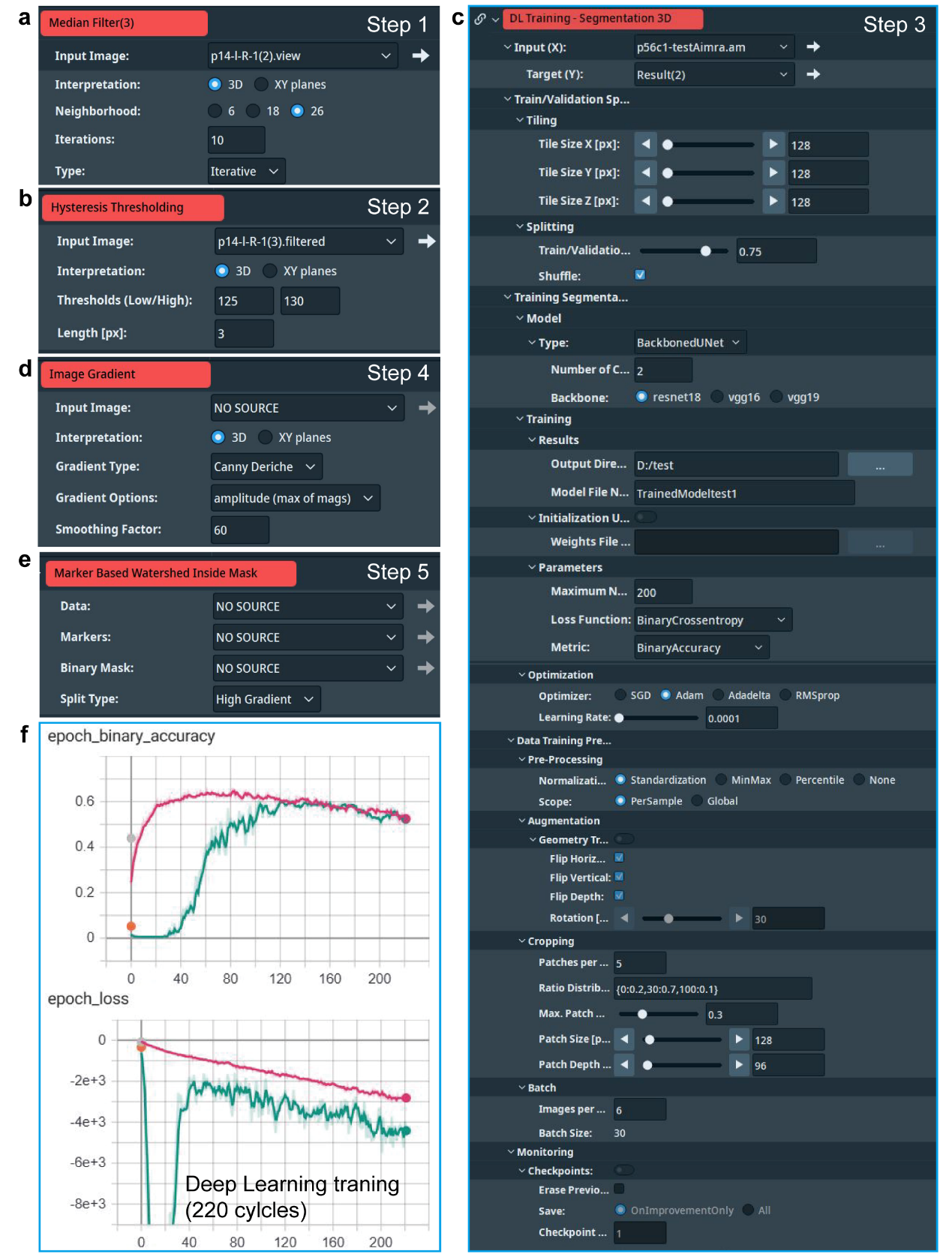


Figure S2. Parameters of the commands used in the soma segmentation process. (a) Using the *Median Filter* command to erode the dendrites and axons. (b) Using the *Hysteresis Thresholding* command to convert the grayscale image into a binary image. (c) Using the *DL Training-Segmentation 3D* command to enhance the accuracy of soma boundary extraction. (d) Using the *Image Gradient* command to facilitate the delineation of boundaries between the adjacent MNs. (e) Using the *Marker-based Watershed inside Mask* command to segment individual MN. (f) The initial deep learning training was generally set to 100-200 training cycles, in order to facilitate parameter tuning. The accuracy and loss function for each training cycle during the initial training were shown here. The magenta curve indicated the accuracy and loss values obtained on the training set, while the green curve indicated the accuracy and loss values obtained on the validation set.


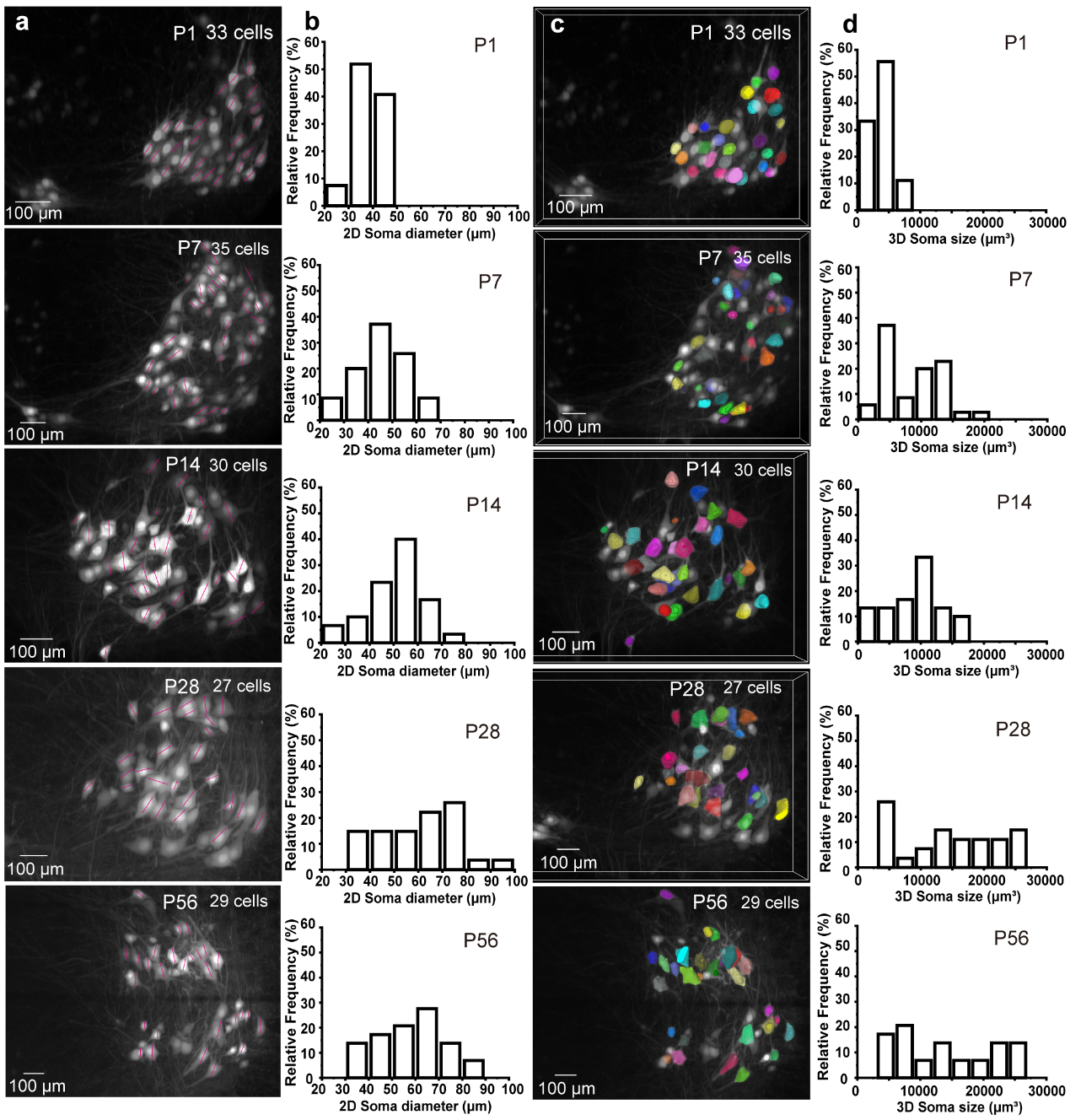


**Figure S3.** Frequency histograms of 2D cell diameter and 3D soma size of MNs during development. (a) 2D cell diameters obtained from z-axis projections of fully contained MNs in approximately 100 µm-thick spinal cord sections at developmental stages P1, P7, P14, P28, and P56. Scale bar: 100 µm. (b) Frequency histograms corresponding to the cell diameter measurements shown in (a). (c) 3D soma sizes of MNs shown in (a), derived from the identical 100 µm-thick sections at developmental stages P1, P7, P14, P28, and P56. Scale bar: 100 µm. (d) Frequency histograms corresponding to the 3D soma size measurements shown in (c).


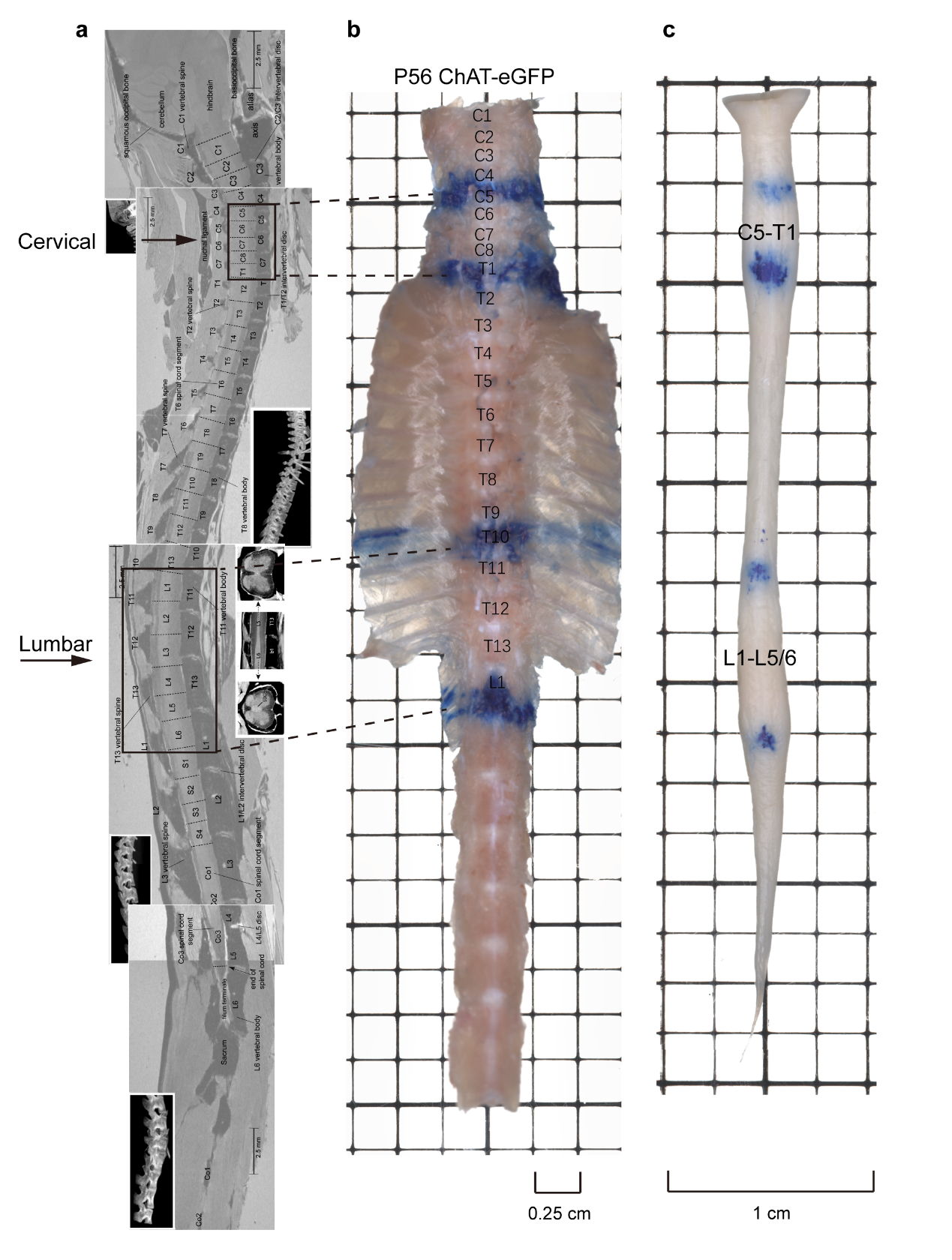


**Figure S4.** The cervical (C5-T1) and lumbar (L1-L6) cord of P56 ChAT-eGFP. (a) Annotated sagittal magnetic resonance image of spinal cord, from Charles Watson et al. in 2012. (b) The relationship between each spinal cord segment and the spines of the vertebrae (Ventral view). (c) The cervical and lumbar cord of P56 ChAT-eGFP (Dorsal view). The length of each square grid is 0.25 cm.


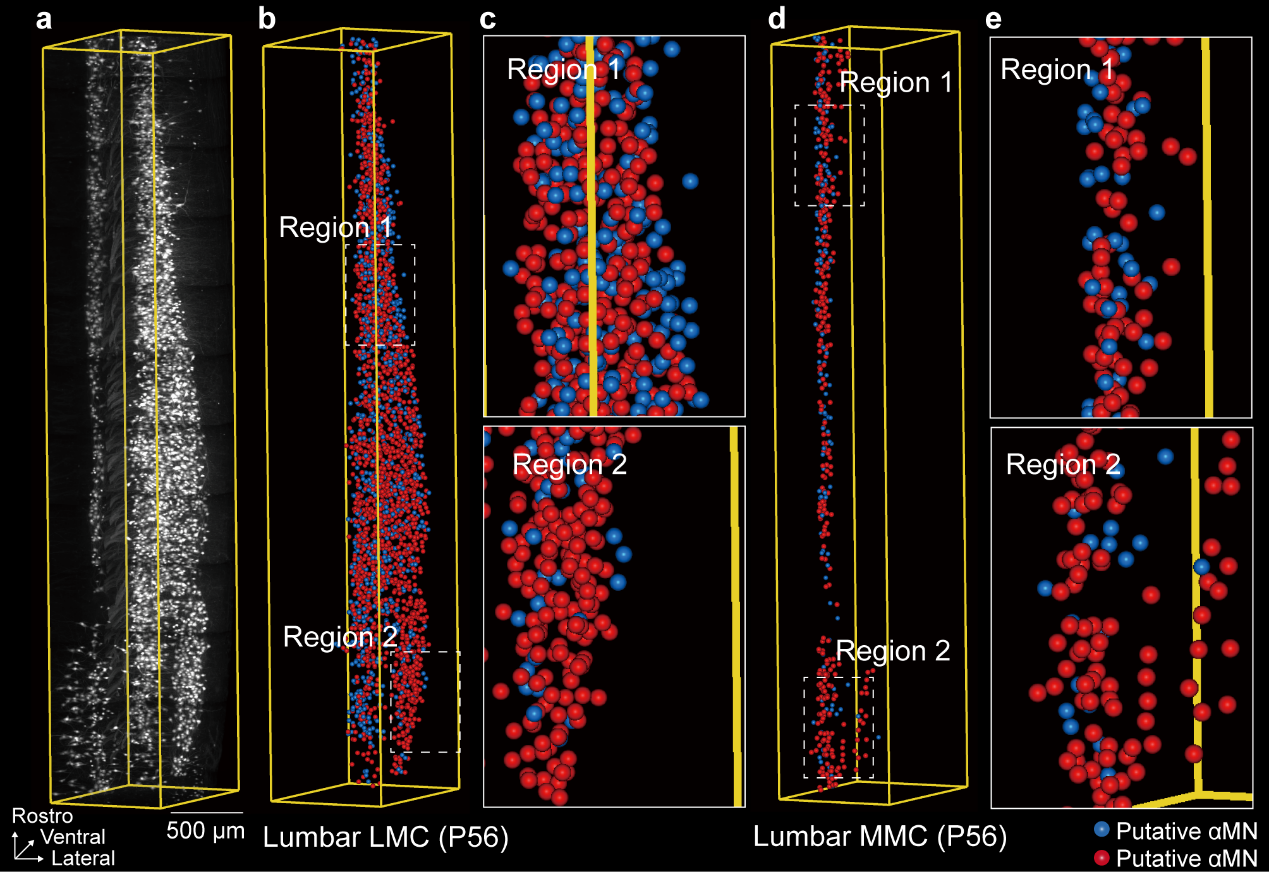


**Figure S5.** Spatial distribution pattern of putative γMNs and αMNs in the lumbar cord. (a) Grayscale image of the lumbar MNs of P56 ChAT-eGFP, with a scale bar of 500 μm. (b) Spatial distribution pattern of the putative γMNs and αMNs of the lumbar LMC. (c) A magnified view of two regions in (c). (d) Spatial distribution pattern of the putative γMNs and αMNs of the lumbar MMC. (e) A magnified view of two regions in (d).


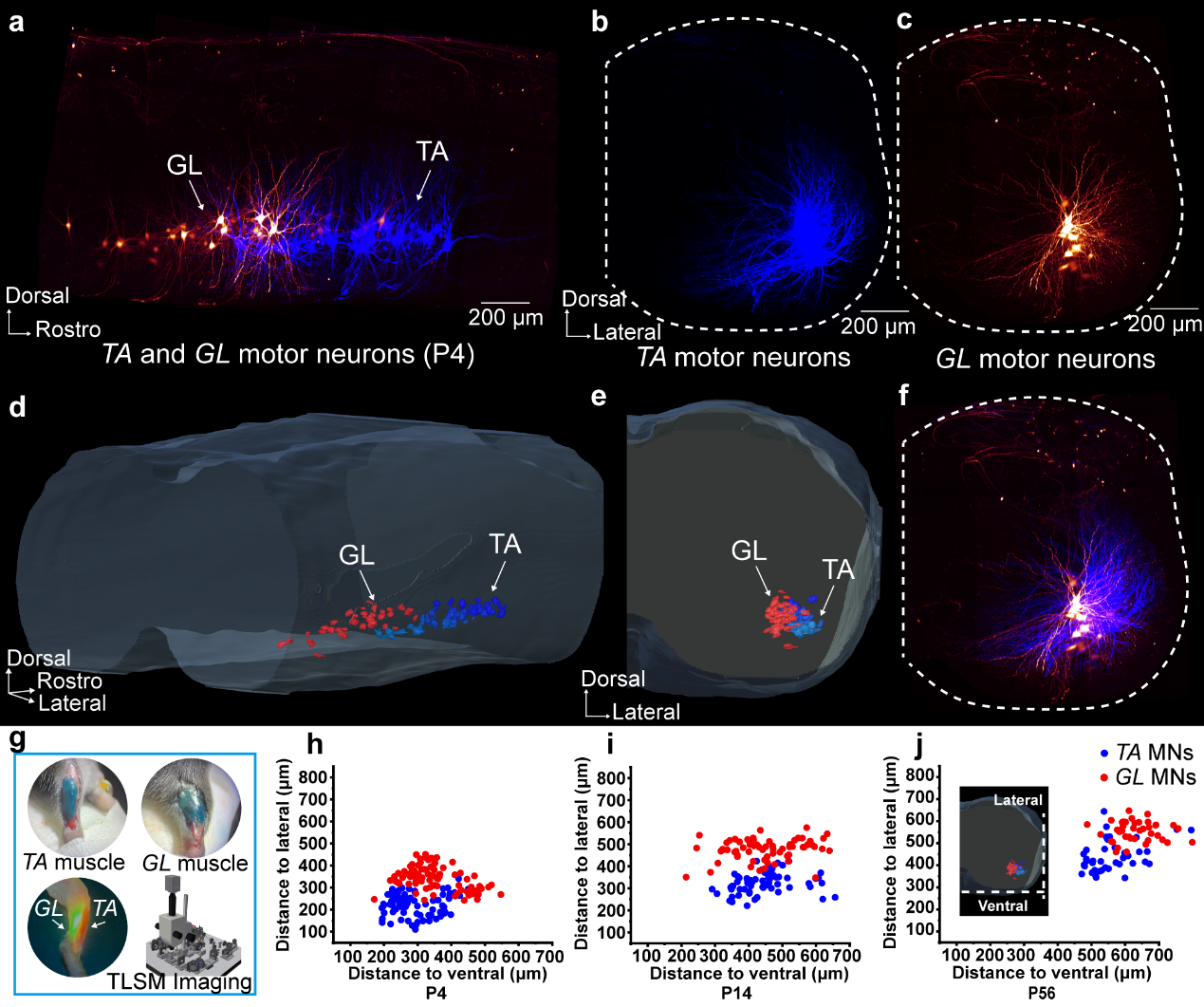


**Figure S6.** Spatial distribution of MNs innervating TA and GL muscles during development. **(A)** The MNs innervating the TA and GL muscles were retrogradely labeled with AdV-eGFP and AdV-tdTomato in the sagittal plane at P4, with a scale bar of 200 μm. **(B) The** TA MNs were labeled with AdV-eGFP in the transverse plane, with a scale bar of 200 μm. **(C)** The GL MNs were labeled with AdV-tdTomato in the transverse plane, with a scale bar of 200 μm. **(D, E)** The reconstructed somas of TA and GL MNs. **(F)** The merged images of TA and GL MNs in the transverse plane, with a scale bar of 200 μm. **(G)** The target muscles are illustrated in pre- (top panel) and post-injection states (bottom panel). **(H-J)** The vertical distance of MNs from the ventral and lateral edges of the spinal cord at P4, P14, and P56. A total of 88 TA MNs and 88 GL MNs were segmented from 3 animals at P4. A total of 64 TA MNs and 64 GL MNs were segmented from 3 animals at P14. A total of 40 TA MNs and 40 GL MNs were segmented from 3 animals at P56.


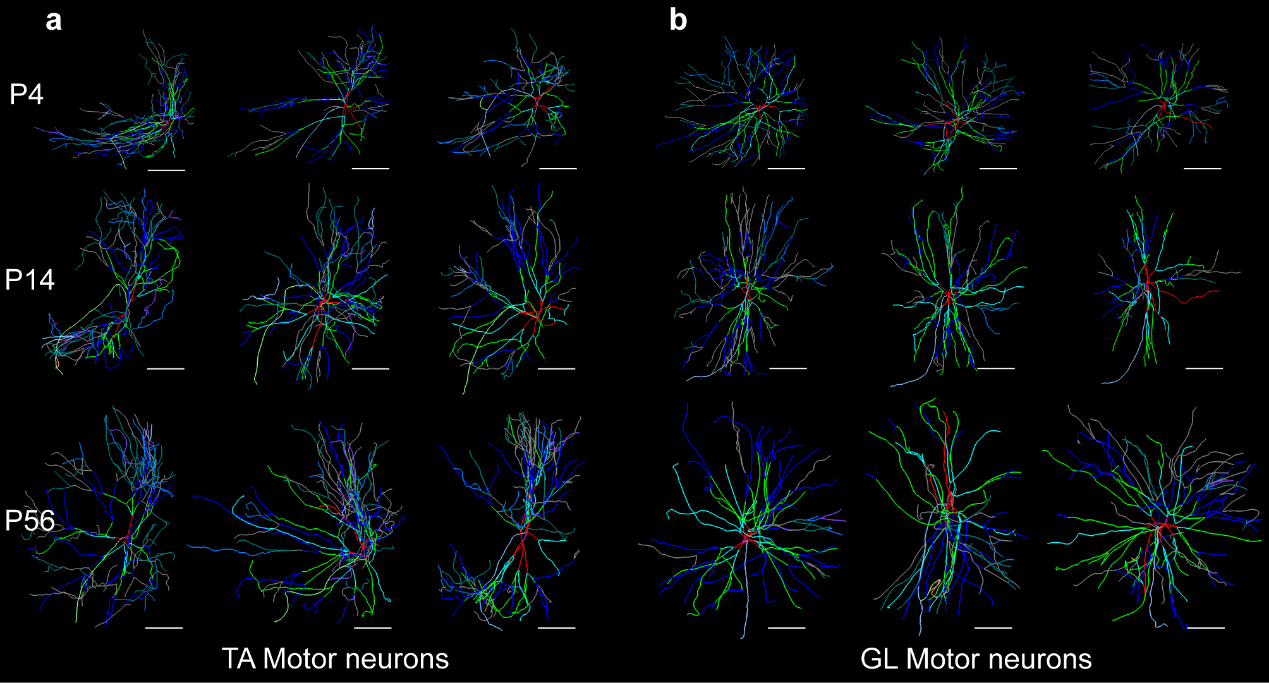


**Figure S7.** The skeletonized dendrites of TA and GL MNs. (A) The three reconstructed TA MNs in the transverse plane at P4, P14 and P56, with a scale bar of 100 μm. (B) The three reconstructed GL MNs in the transverse plane at P4, P14 and P56, with a scale bar of 100 μm.


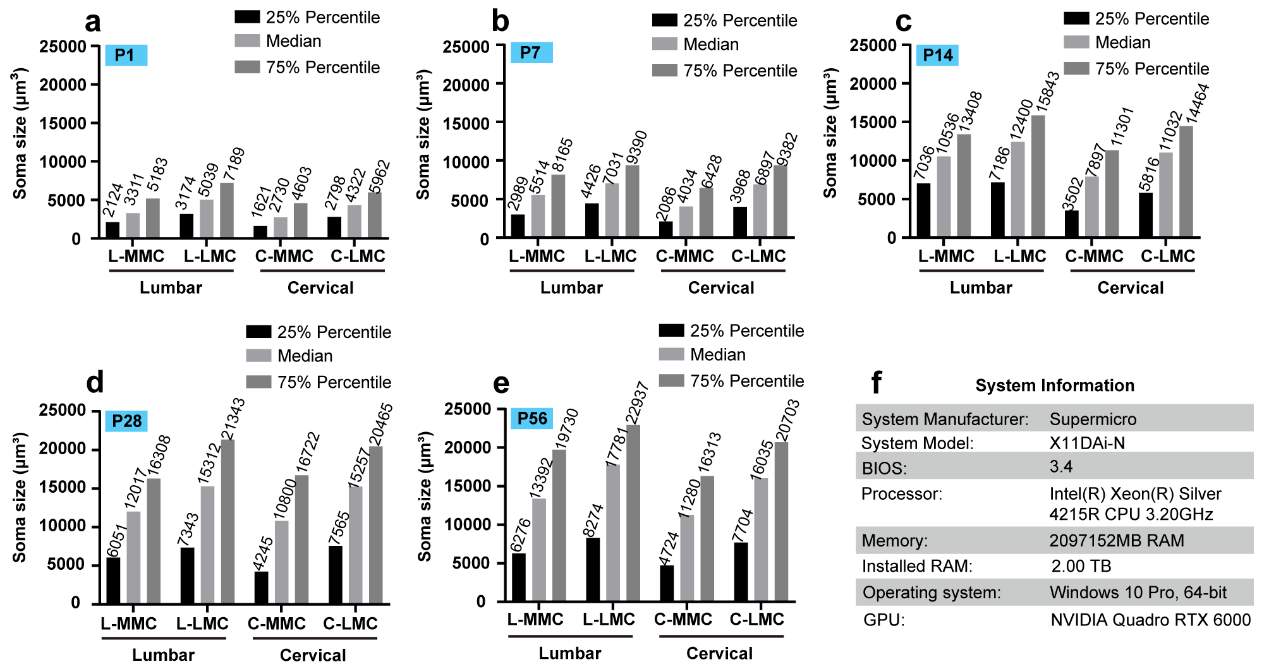


**Figure S8.** Soma size of MNs during postnatal development at the 25% percentile, median, and 75% percentile. (a) The 25% percentile, median, and 75% percentile of the soma size of MNs at P1. (b) The 25% percentile, median, and 75% percentile of the soma size of MNs at P7. (c) The 25% percentile, median, and 75% percentile of the soma size of MNs at P14. (d) The 25% percentile, median, and 75% percentile of the soma size of MNs at P28. (e) The 25% percentile, median, and 75% percentile of the soma size of MNs at P56. (f) Hardware setup of the workstation.
